# Exploring the barriers to Pap smear test compliance: A qualitative study for improving cervical cancer screening in the primary health care

**DOI:** 10.34172/hpp.42485

**Published:** 2024-03-14

**Authors:** Mansoore Shariati Sarcheshme, Mehrsadat Mahdizadeh, Hadi Tehrani, Mohammad Vahedian-Shahroodi

**Affiliations:** ^1^Department of Midwifery, Neyshabur Branch, Islamic Azad University, Neyshabur, Iran; ^2^Social Determinants of Health Research Center, Mashhad University of Medical Sciences, Mashhad, Iran; ^3^Department of Health Education and Health Promotion, School of Health, Mashhad University of Medical Sciences, Mashhad, Iran

**Keywords:** Cervical cancer, Cancer screening, Pap smear, Healthcare provider, Qualitative research, Perception, Barrier

## Abstract

**Background:**

Cervical cancer in Iran ranks as the fourth most frequent cancer among women. Pap smear (PS) is the best standard for detecting cervical cancer, but many people, even healthcare providers (HCPs), do not maintain it. HCPs play a critical role in promoting PS uptake. The purpose of the study was to explore barriers to cervical cancer PS screening compliance from the HCPs’ perspective.

**Methods:**

The present qualitative content analysis was conducted through semi-structured in-depth interviews. A total of 28 HCPs were interviewed between July and August 2020. A diverse sample of HCPs was selected using purposive sampling. Data analysis was based on the five steps proposed by Graneheim and Lundman. MAXQDA (2020) was used for data analyzing.

**Results:**

Ten key sub-categories were identified and organized into three categories: individual, environmental, and socio-cultural factors. The sub-categories included inadequate risk perception, inappropriate attitude, low commitment, emotional factors, low priority over health, requirements and consequences of the test, deficiencies of health centers, organizational factors, traditions and religious believes.

**Conclusion:**

HCPs face multiple barriers for PS. Exploring and decreasing barriers of PS in HCPs may increase compliance in them and their clients because they play an influential role in instructing and persuading women to take the PS. There is need to explore these barriers and identify possible interventions to change them. Insights from this study are useful for developing policies around national PS programs, too.

## Introduction

 Cervical cancer is a significant health problem for women worldwide, especially in developing countries. As per the GLOBOCAN 2020 report, it is the fourth most frequent cancer in women on a global scale,^1^ yet is predicted to rise to the first rank by 2030.^2^ Half of all women who die from cancer worldwide have cervical cancer,^3^ and most of them (80%-85%) live in low- and middle-income countries.^3^ Developing regions will face almost two-third of the cervical cancer cases predicted for 2050.^4^

 In Iran, cervical cancer is also the fourth most common cancer among women, with an age-standardized incidence rate of 2.5 per 100 000 people in 2020, up from 2.17 in 2009.^5,6^ The average age of diagnosis for Iranian women is about ten years younger than the global rate ^2^.

 The Pap smear (PS) test has been around in the Iranian health system since 1989, but various studies show this test has not been favored even by healthcare providers (HCPs), and few people go for it.^7-11^ The HCPs who should be responsible for opportunistic cervical cancer screening of women they care for, are not keen on getting screened themselves.

 HCPs have various qualities that can help promote PS. They work in different parts of the health system, have direct contact with society, constitute a large part of the country’s female population, and play an influential role in instructing and persuading women to take the PS.^10^ They can also be role models for women and help advance PS if they show a positive attitude and behavior. HCPs’ non-compliance to policies regarding PS is against not only the country-specific objectives but the international policies.

 Many cross-sectional quantitative studies have been conducted on the knowledge, attitude, and practice of HCPs about the PS. They reported low compliance with PS because of time pressure, absence of symptoms, embarrassment, fear of pain, carelessness, unwillingness to be examined by colleagues, and lack of scientific belief in screening methods.^7,8,10^

 Qualitative research seeks to explore the beliefs and attitudes that affect each other and result in a specific phenomenon.^12^ In a web-based search, no qualitative research was found to explore the viewpoints and causes of poor adherence to PS in HCPs in Iran. Then, the researchers decided to conduct qualitative research to explore HCPs’ perspectives on barriers to cervical cancer PS screening compliance

###  Materials and Methods 

###  Study design and participants

 A conventional qualitative content analysis was done to identify the barriers to compliance with the PS in HCPs. Sampling was performed from HCPs working in comprehensive health service centers in Mashhad, one of the religious cities of Khorasan Razavi province, Iran. The participants were selected through a purposive sampling method with maximum diversity of age, education level, work experience, duration of marriage, and history of PS. The sampling continued until data saturation. Out of the 30 HCPs, who consented to the interview, six did not have enough time to complete the interview; thus, they were excluded from the study. The participants included four general practitioners, two nurses, and eighteen health workers (5 public health experts, 3 health educators, and 10 midwives).

 Before the interview, the aim of the study was explained to HCPs, and if they consented to participate in the research, a written informed consent form was signed according to the Helsinki’s Declaration.

 The inclusion criteria were: holding a degree in a health-related field (such as public practitioner, midwifery, nursing, public health, or health education), femininity, being married, at least three years of sexual experience, and willingness to participate in the research. Those who were not willing to take part in interviews or continue cooperating with the researcher were excluded from study. There was no relationship with participants established prior to study commencement.

###  Setting

 Participants were interviewed at a private room in the comprehensive health centers. Before the main interview, two preliminary interviews were held with individuals from different groups, who were not the main research subjects. The purpose was to evaluate the validity of the data collection instrument and find any potential bias or fault in the interview process for the research team. The first author, a Ph.D. student of health education, conducted the interviews using a face-to-face semi-structured in-depth interview and after 22 interviews, theoretical saturation was obtained. But for more certainty, two more interviews were conducted to identify new codes. The data collection finally ended with 24 interviews. The average time of interviews were 35-45 minutes depending on the participant and interviewee interaction.

 At the beginning of the interview, some general and open-ended questions were asked. Guided questions, according to the result of a primary literature review, were used to conduct the interviews, followed by probing questions such as “What do you mean?”, “Why?”, “Please explain more.” Some examples of guided questions are provided in Box 1.

**Box 1. Samples of guided questions in interviews**

1. Have you ever going for Pap smear?

• If yes, did someone suggest you to go for that test? What factors motivate you to do Pap smear?

• If no to question no 2: Why did you not go? What barriers and challenges have you faced to perform Pap smear?

2. How do you think the organization in which you are working can be effective in motivating you to do a pap smear?

3. As a health personnel, if you want to plan to improve your cervical cancer screening behavior, what strategies do you suggest?

**Probing questions**: What do you mean? Why? Please explain more.

**Closing question**: Do you have any question, requests/suggestions?

###  Data collection and analysis

 Concerning the qualitative data analysis, the researchers employed the five-step approach proposed by Graneheim and Lundman (2004),^13^ in MAXQDA (2020). At the first step, the interviews were recorded by an audio recorder and immediately transcribed verbatim, which served as the primary data of study. The researchers immersed themselves in data by listening to the recorded voices and reviewed the manuscripts frequently, finally divided the textual content into semantic units, divided intensive semantic units based on explicit and implicit concepts in statements to convey significant meanings, followed by coding and abstracting data. The basic coding structure and topics were discussed by two authors who coded the first three transcripts. Differences in coding were discussed and the final framework was created.

 This step of analysis also involved the confirmation of accuracy of codes by supervisors and research team. In the fourth stage, researchers assigned codes to singular topics based on similarities and differences in meaning to form sub-categories, and finally, grouped sub-categories at a higher level of abstraction, thereby categorized them and ultimately ascertained the main categories. The data were analyzed at the same time of data collection, which enabled the researchers to detect emerging codes and themes. Demographic information checklist along with field notes (made after interview) were used for data collection too.

###  Rigor

 To confirm the validity and strength of study, the criteria suggested by Lincoln and Guba were used.^14^ These criteria provide a systematic approach for assessing the trustworthiness and rigor of qualitative research studies. To establish credibility, member-check was used to seek feedback from three participants and another authors. Adequate time was allowed for data collection, interpretation and long-term interaction with the data. Another important way to increase credibility is high credit of researcher. For this reason, the interviews were held by one of the authors, a Ph.D. student of health education with an M.S. degree in midwifery. To increase transferability, the purposing sampling with principle of maximum diversity of participants was adhered to in data collection, and thick description was used by describing the research process in detail. Also, through providing appropriate quotes and explaining the participants’ opinions, the transferability was increased. For confirmability, we used of the practicing reflexivity and confirmability audit. The researcher did not interfere with her preconceptions in the process of data collection. The three authors reviewed and validated the participants’ coding steps and citations, recorded all research details and took notes on each step of the work. Dependability was achieved by providing clear and transparent documentation of procedures of study, data collection, analysis, and theme extraction. The whole procedure of study was reviewed by an audit to confirm the accuracy.

## Results

 Ten subcategories and three main categories including individual, environmental, and socio-cultural factors, emerged from analysis of data. (Table 1). The most frequent sub-categories included negative attitude, low priority over health, low commitment, organizational factors, and culture. Although the participants were health service providers, but 21.1% of the them had never had any experience of the PS. Some demographic characteristics and PS history of participants is reported in Table 2.

**Table 1:** Themes, sub-themes, and semantic units of non-compliance with PS in HCP

Category	Sub-category	Semantic code
Individual factors	Inadequate risk perception	False confidence Not taking the risk seriously Not having risk factors
	Poor awareness inappropriate attitude and behavior	Lack of motivation Improper performance Inappropriate attitude Poor awareness
	Low acceptance and commitment	Wrong belief Lack of commitment to test
	Low priority over health	Prioritizing other important priorities Not valuing your health Weakness in time management
	Psychological and emotional factors	Previous negative experience Modesty Fear Disliking gynecological examinations
Environmental factors	Requirements and consequences of the test	Necessary preparations for sampling Adverse consequences of sampling Costs
	Deficiencies of health centers	Inappropriate physical environment Low sampling quality Late delivery of results Lack of access to reliable centers
	Organizational factors	executive problems Policies Lack of positive reinforcement
Sociocultural factors	traditions	Sacrificing and extreme motherhood Extreme restraint
	Religious believes	Family taboos Culture of obligation and coercion False religious beliefs

**Table 2:** Demographic characteristics and Pap smear history of participants

**Variable**	**Mean ± SD**
Age	40.57±9.7
Gravidity	1.1± 2.1
Parity	4.4±3.1
Live children	0.97±1.94
Marriage age	3.8±24.2
Marriage duration	10.5±16.6
Menarche age	1.3±12.9
work experience	9.3±14.3
First PS after marriage	2.6 ± 3.2
Number of PS	2.4± 2.6

###  Individual factors

 This category comprised five sub-categories as follows:

####  Inadequate risk perception

 Some participants perceived themselves invulnerable to cervical cancer due the absence of any risk factors such as vaginal symptoms. This misguided sense of confidence hindered HCPs’ willingness to take the PS.

 “*I did not do it because I had no problem. For example, this bad-smelling secretion, I do not have any excessive secretion, itching, or irregular periods” *(No. 24, B.S. in Public Health, 35 years old).

####  Poor awareness, inappropriate attitude and behavior

 Inadequate and inaccurate information about the cervical cancer, as well as the PS, unfavorable attitude, and lack of motivation were barriers to the performance of PS.

 “*In my opinion, many of those afflicted with cervical cancer are those who are not committed to their relationships and have increased extramarital relationships that may cause most of those cancers” *(No. 20, B.S. in Public Health, 50 years old).

####  Low acceptance and commitment

 Misbelief about cervical cancer and lack of commitment to the performance of PS, were two essential reasons HCP’ uninterest in PS. Laziness, negligence, making excuses, and recurrent ignorance were signs of low commitment to PS.

 “*I also observe some personal hygiene very much. For example, I felt that because I am preventing, I probably will never get sick until the end of my life” *(No. 18, B.S. in Public Health, 42 years old).

####  Low priority for health

 Some participants reported prioritizing different aspects of lives over their health, including domestic chore, childcare, and work. Thus, they did not spend enough time on the PS.

 “*That is because in our priorities, the time we spend for others is more valuable than the time we spend for ourselves. On our mind, the priority goes to children and husband” *(No. 8, General Practitioner, 50 years old).

####  Psychological and emotional factors 

 Participants mentioned some psychological factors as barriers for PS, such as fearing the pain of internal examination and sampling, unfavorable test results, embarrassment with the sampling position and revealing intimate organs, and having negative experiences.

 “*Another issue is that we do not want to know and are afraid of reality. We are worried if we have a problem, we may not accept it. Usually, this fear exists in those already in the informed groups” *(No. 1, Midwife, 51 years).

###  Environmental factors

 This category consisted of three sub-categories, as follows:

####  Requirements and consequences of the test 

 The necessary preparations for sampling, such as no intercourse three days before, and the limitation of suitable days of the menstrual cycle for sampling can delay and sometimes stop the PS. Few adverse effects of sampling, such as spotting or infection after the test, were also among other factors mentioned by the participants.

 The cost of PS (financial cost and time) included the cost of a liquid-based type of PS, the cost of visiting a gynecologist, making an appointment with the doctor, and waiting in doctor’s office were other problems that were mentioned.

 “*Because of its specific conditions and that we had to do many things before the test, I never decided to go for it.” *(No. 7, Public Health Expert, 30 years old).

 “*I had to make an appointment at least ten days in advance for a PS in a private clinic, then I waited for three hours for my turn, and then I had to accept a tenfold cost of a regular PS to get the test result.” *(No. 12, B.S. in Public Health, 34 years old).

####  Deficiencies of health centers

 The inappropriate physical environment of sampling rooms with old and low-quality equipment, using old method which lacks accurate results, inappropriate and insufficient sampling by non-experts, late PS results, and the lack of access to reputable laboratory centers were some problems in health centers mentioned by HCPs.

*have been working in the centers in 99.5 percent of cases, the result is the same showing no problem, it writes category one, category one, category one. A person doubts a little. Well, it raises doubts.” *(No. 15, Midwife, 45 years old).

####  Organizational factors

 Numerous problems and disorders at higher levels of health management are among the important factors the participants mentioned. Work-related issues within the environment of health centers, such as work load due to the poly valent plan, lack of staff compared to the covered population, self-completion of electronic health records by employees, and lack of in-service training were among the mentioned barriers to the performance of PS.

 “*I’m dead on my feet. I mean, since we became polyvalent, we were under much pressure and suffered a lot” *(No. 6, Midwife, 54 years old).

 “*Everybody is considered among those covered by him/herself because we mostly access the records. For me myself, for example, my family are covered by my own record. For me, the PS record is still vacant” *(No. 22, Midwife, 36 years old).

 Participants pointed out that some of the existing policies, such as the lack of PS in the cohort health assessment plan for HCPs, unavailability of supplementary insurance for all HCPs, insufficient insurance coverage of PS expenses, and absence of a regular health checking system hindered the implementation of PS. They suggested that positive incentives such as the inclusion of points in yearly evaluations, and using a notification system (SMS or email) can enhance PS performance.

 “*Like this staff monitoring plan that is being implemented, everything is there except for this. I mean the cohort plan as you know” *(No. 16, Nurse, 37 years old).

 “*If we have time in the morning to go for a PS, we do not need to take an absence of leave. So, it can encourage us” *(No. 16, Nurse, 37 years old).

###  Socio-cultural factors

 This category consisted of two sub-categories, as follows:

####  Traditions

 Traditions and culture of society encourage women and mothers to adhere to their motherly role. Self-sacrifice and excessive attention to family health have caused women to think less about their health, not spend enough time on their own health needs, and always consider the welfare, comfort, and health of others as their main duty.

 “*That’s the matter of traditional upbringing. Our mother sacrificed herself for our education, for our health, for preparing us a good meal to eat when we came back from school. She did her best for us to sleep and study well and the like stuff. We inherited all this from her. Now, we take exactly the same route in life.” *(No. 21, Midwife, 44 years old).

####  Religious believes

 Some incorrect religious beliefs have also caused some people not to consider screening necessary and not to go for it.

 “*From a religious point of view, it is problematic, and I should not go for examination until I have a specific problem. Because you have to be examined, and this examination is sinful from a religious point of view” *(No. 15, Midwife, 45 years old).

 Some HCPs believed that the culture of obligation and coercion can lead to PS screening being mandatory. It can make them adhere more to the recommended screening guidelines and not only perform PS themselves but also suggest it to all eligible patients.

 “*Well, I think for us, the Iranian, our culture is such that we have to be obliged to do something. Otherwise, we never do the right thing as required” *(No. 21, Midwife, 41 years old).

 “*If they set a rule for having the PS test and having to submit the result by a certain date, everyone will go for the test” *(No. 19, Midwife, 31 years old).

## Discussion

 Numerous studies have shown the importance of HCPs as predictors of cervical cancer screening use.^15^ Thus, understanding their perspectives on the barriers of performing PS plays a significant role in promoting this preventive behavior. In this qualitative study we explored that the HCPs are not keen on performing the PS and their barriers were categorized in three main themes: individual factors, environmental factors, and sociocultural factors.

 Several individual factors prevented HCPs from performing regular PS. Low perceived risk is a significant barrier to HCPs performing regular PS. They believed that they were not susceptible to the disease and were not at risk of cervical cancer due to the lack of clinical symptoms or risk factors. This led to their false self-confidence and not performing the PS.

 In the study was conducted by Karena and Faldu, 87.6% of the nursing staffs had never taken a PS, due to some reason such as not being at risk, not having symptoms, feeling shy or uncomfortable, afraid of the results.^16^ In the study of Mutyaba et al, 65% of hospital medical staff did not consider themselves susceptible to cervical cancer and 81% had never been screened.^17^ Mohammadi et al reported that the most important reasons for not performing the PS in health workers in Isfahan were lack of problems in the reproductive system, reluctance to take the test, lack of self-care, and awareness.^3^ These results have also been mentioned in other studies too.^18,19^

 Limited knowledge and low perception of the importance of PS screening and its guidelines is a key factor in HCPs not prioritizing screening for themselves or their patients. Mohammadi et al pinpointed the lack of problems and low awareness as the main reasons for not performing the PS in Chaharmahal and Bakhtiari staff.^3^ In the studies of Heena et al and Roux et al, many health service providers also lacked sufficient knowledge of cervical cancer^9,20^ and, therefore, had not gone for the PS. Contrary to these studies, the mere knowledge of the risk factors does not necessarily lead to increased screening behavior, as evidenced by Yörük et al among Turkish nurses and midwives. Despite being familiar with risk factors, only 35% of them had a history of the PS.^21^

 The results showed that having a positive attitude towards health and prioritizing prevention over treatment are important factors in promoting PS performance among HCPs. Fallahi et al considered prioritizing health and developing a positive attitude towards it, as effective factors in performing the PS.^2^ In line with these results, a study conducted on 1900 health workers in Isfahan showed that the attitude of 42.15% was negative or neutral.^3^ In the study of Singh et al, even though nurses were highly aware of cervical cancer screening, their attitude was weak.^22^

 Another barrier was low commitment, which occurs when people have a vague understanding of the values and goals. Lack of commitment recurrently delayed the performance of PS. Lack of commitment to performing the test for reasons such as laziness, neglect, carelessness, inaccuracy, and not giving importance to the test, caused repeated delays in performing PS.

 Mahalakshmi and Suresh reported negligence and carelessness about their health as the barrier of PS performing.^23^ Other studies also mentioned barriers such as inattention and carelessness.^11,21,24-27^ The use of reminders (such as SMS or email) or making appointments can be effective strategies to reduce this barrier.

 Recommendation by a significant person can increase motivation, reduce anxiety or fear, and increase perceived social support for this behavior. As the present findings showed, ignoring the advice of significant people can be a barrier to preventive screenings. As reported by the American Cancer Society, the most important factor in creating motivation for screening was the recommendation by doctor or health staff.^9^ In different studies, reminders and recommendations by health staff, and encouragement by friends, relatives, family members, and colleagues were mentioned as motivating factors.^9,24,26,28-31^

 The present results showed not prioritizing health and having other priorities was a significant barrier to performing the PS. Darj et al considered paying attention to the body and health as motivational factor for performing the PS.^32^ An incentive for Guatemalan and Thai women to perform the PS was their interest in health and desire to know about their health status.^33,34^

 The time-consuming nature of visiting a doctor and performing the PS is a common barrier mentioned in this study. Many studies have shown that women are often involved in domestic chore, childcare, and work, leading to a lack of time for prioritizing their own health,^23,30,32^ and therefore not going for the PS.^35,36^ This barrier has been mentioned in several studies.^7,9,26^ Since the facilities and doctors are available at health centers for HCPs, other factors may be more effective in not performing the PS.

 Psychological factors such as the fear of cancer, medical processes, and the pain of sampling process are deterrents of PS.^23,32,37,38^ In general, fear of medical processes can cause avoidance or delay in performing the PS. This fear prevailed in people who had negative experiences of medical methods. Fear of pain during the sampling process, possible positive test results, and false diagnosis were also among the reasons that the participants mentioned, too. The results of this study are consistent with many studies that have raised fear as a barrier to performing PS.^22,25,30^

 Feeling embarrassed and ashamed, due to internal examination, is a reason for fewer visits for the PS. Embarrassment of revealing intimate organs and the position required for sampling, and embarrassment of internal examination, especially examination by colleagues, were among the important issues that were repeatedly mentioned. The results of Yusuf’s study of physicians showed that 75% of them had never performed the PS in their life, and among the reasons were lack of time and modesty.^39^ Turkish nurses and midwives also cited indifference, fear, and embarrassment of performing PS as the reasons for less PS. This barrier has been mentioned in many studies as well.^7,10,22,25,27^

 As mentioned by our participants, negative previous experiences act as barriers to performing the PS, affecting people’s willingness to repeat the screening.^23,40^ Roux et al also mentioned the perceived quality of care and satisfaction or dissatisfaction with previous screening as a factor affecting the willingness or unwillingness to repeat the PS.^20^

 Factors like limited appropriate time for PS during the menstrual cycle, required prior preparations, and mild complications of the test deter HCPs from performing PS. Despite the availability of sampling facilities at work, HCPs are prevented by inappropriate physical environment in health centers, low quality of sampling, distrust in results, and long delays in providing test results. Some HCPs mentioned that they could not go to labs and private centers outside work hours because they worked long and had a lot to do. Several studies mentioned the lack of laboratory facilities, inadequate working staff and resources, and late preparation of test results as the causes of less willingness to perform cervical cancer screening.^20,32,33^ Easy and cheap access is an important motivator of performing the PS,^41^ but increasing access to services does not always increase their use,^20^ as the results of the present study showed.

 Lack of support and attention of the health system to employees’ health, including inadequate in-service training, lack of contracts with reputable laboratories, inadequate incentives, exclusion of PS in the cohort plan for monitoring employee health and executive problems are important factors in not performing the PS. As HCPs mentioned, creating motivation in different ways and using legislative levers can improve the PS.

 HCPs admitted that they did not care about maintaining and improving their health until they faced a pressing factor like signs of illness, losing their work or status because of the disease, or having compulsory yearly exams by the institution. Mahalakshmi et al also pinpointed the change in government policies based on making PS mandatory.^23^ Although mandatory screening can increase screening rates, forcing healthcare professionals to perform screenings violates their autonomy and privacy rights.

 Providing financial resources and legal support is essential for implementing organized screening programs. Many studies have cited the financial costs of PS as a barrier to doing so.^2,20,23,30,32,33,37,38,40^ Australia, which is the first country ready to eliminate cervical cancer, allocated 220 million Australian dollars to create and manage the national cancer screening registry in 5 years.^15^ Financial costs are not a significant barrier for all HCPs in this study due to access to doctors, midwives, and supplementary insurance.

 As the participants mentioned, socio-cultural factors such as extreme role of motherhood and excessive modesty act as barriers to preventive screenings. The cultural and social pressure promotes an ideal mother who is versatile and selfless, responsible for all aspects of children’s lives. Extreme motherhood often leads to sacrificing the well-being and personal goals of mothers due to gender stereotypes and unfair burden on women. This was a new code that other studies did not report such barriers, possibly due to the specific culture of Iranian women that encourages Self-sacrifice and excessive motherhood.

 Excessive modesty in discussing sexual issues can be a result of cultural or religious beliefs that consider such topics as taboo or sinful. Shame and secrecy around sexual issues can lead to misinformation, confusion, and negative attitudes. According to Darj et al, Nepalese women believed that discussing the reproductive system was embarrassing and largely avoided.^32^ Breaking the taboo around sexual health requires changing attitudes and cultural norms, which takes time and effort. Some HCPs had false religious beliefs that hinder necessary tests and interventions. These false religious beliefs are interpretations of religious instructions that are not supported by religious texts or traditions. These beliefs may be based on personal or cultural biases, misinterpretation of religious texts, or the influence of non-religious factors such as politics or social norms. According to the results of Marashi and colleagues’ study, some religious instructions and social norms strongly prohibit women from showing their intimate organs even to doctors.^42^ Belief in fate and destiny can affect preventive behaviors,^35,42^ with some people believing that certain diseases are destined and cannot be prevented. This belief that disease and healing are from God has been recognized as a barrier to preventive intervention for cervical cancer.^40^ In Abdi and colleagues’ study, Pakistani and Somali immigrants living in Norway believed that Muslims do not get afflicted with this disease.^43^

 Various factors may account for the discrepancies in the outcomes of different studies, such as the setting of study, different healthcare systems, access to facilities, and participants’ cultural, social, and economic backgrounds, their age, education, and health attitudes.

## Limitations

 There were a number of limitations in this study. It was conducted in only one city in Iran with a particular context, which limits the generalizability of findings. Although focus group discussions could have provided more in-depth insights, they were not feasible due to restrictions caused by the COVID-19 pandemic. Therefore, face-to-face interviews were used to collect the required data.

## Conclusion

 Understanding the barriers that hinder the use of PS is essential for increasing its overall reception. Health care providers not only play a key role in providing PS screening services, but also educating and motivating women to undergo the test. Therefore, HCPs’ perspective can help to identify the gaps and challenges in the delivery and quality of cervical cancer screening services, and design effective and tailored interventions to improve PS screening. Results of the study are helpful for policy makers to creating policies in order to promote national cervical cancer screening programs as HCP_s _mentioned: Planning educational interventions to change the beliefs and attitudes, creating motivation, reviewing managing planning and national policies, improving the quality and quantity of services in health centers, and promoting a correct health culture.

## Recommendations

 Researchers gave a number of suggestions to increase HCPs’ participation in performing the PS, based on their opinions:

 The suggested behavioral changes (educational, motivational) are:

- In-service training category: Providing the latest scientific information on cervical cancer and PS to eliminate knowledge gaps, changing attitudes by introducing cases treated due to timely visits for PS, time management, and career planning categories.
- Counseling: Counseling with a psychologist at comprehensive health service centers to overcome fear and shame.
- Positive reinforcement: Awarding points in evaluation, providing incentive leave for regular test takers, and allowing hourly leave for personal visits for PS.
- Negative reinforcement: Removing points in case of not taking the test (deduction from leave, deduction of points, …).

###  Managerial evaluation

- Improving the quality of sampling at comprehensive health service centers: Quality of sampling room, new equipment, upgrading the management of sending samples and receiving responses
- Reducing the cost of testing: Free testing for HCPs or covering part of the costs, contracting with reputable laboratories and sending staff or sending samples over there, using a referral system for testing
- Using the capacity of midwives at health centers to perform the test and contracting with reputable laboratories to send the PS.
- Entering the PS in the cohort health monitoring plan of service providers policymaking.

###  Legislative leverage

- Having the result of PS at the time of employment in comprehensive health centers or creating a health record is mandatory.
- Annual promotions require mandatory testing.
- Regular examinations are essential.
- Regular PS is a criterion for evaluating service providers.
- PS reminders are sent via email and Ministry of Health systems.
- Supplementary insurance provisions are available for all staff.

## Acknowledgments

 We thank the HCP_s_ of the comprehensive health service centers who participated in this research.

## Competing Interests

 The authors declare that they have no competing interests.

## Ethical Approval

 The study has been approved by the Mashhad University of Medical Sciences (ethics code: IR.MUMS.FHMPM.REC.1400.009) and informed consent was got from all participants. Names and identities of the study participants were not used while analyzing data.
